# Drug repositioning for orphan genetic diseases through Conserved Anticoexpressed Gene Clusters (CAGCs)

**DOI:** 10.1186/1471-2105-14-288

**Published:** 2013-10-02

**Authors:** Ivan Molineris, Ugo Ala, Paolo Provero, Ferdinando Di Cunto

**Affiliations:** 1 Molecular Biotechnology Centre, Department of Molecular Biotechnology and Health Sciences, University of Torino, Via Nizza 52, 10126, Torino, Italy

## Abstract

**Background:**

The development of new therapies for orphan genetic diseases represents an extremely important medical and social challenge. Drug repositioning, i.e. finding new indications for approved drugs, could be one of the most cost- and time-effective strategies to cope with this problem, at least in a subset of cases. Therefore, many computational approaches based on the analysis of high throughput gene expression data have so far been proposed to reposition available drugs. However, most of these methods require gene expression profiles directly relevant to the pathologic conditions under study, such as those obtained from patient cells and/or from suitable experimental models. In this work we have developed a new approach for drug repositioning, based on identifying known drug targets showing conserved anti-correlated expression profiles with human disease genes, which is completely independent from the availability of ‘ad hoc’ gene expression data-sets.

**Results:**

By analyzing available data, we provide evidence that the genes displaying conserved anti-correlation with drug targets are antagonistically modulated in their expression by treatment with the relevant drugs. We then identified clusters of genes associated to similar phenotypes and showing conserved anticorrelation with drug targets. On this basis, we generated a list of potential candidate drug-disease associations. Importantly, we show that some of the proposed associations are already supported by independent experimental evidence.

**Conclusions:**

Our results support the hypothesis that the identification of gene clusters showing conserved anticorrelation with drug targets can be an effective method for drug repositioning and provide a wide list of new potential drug-disease associations for experimental validation.

## Background

Orphan diseases (OD) are commonly defined as rare disorders with prevalence of less than 200000 cases in the US or of less than 1:2000 in Europe. Despite the low frequency of the single disorders, OD represent an extremely important medical and social challenge, because the ~ 6000 known rare diseases affect about 10% of the population in developed countries and because only in less than 5% of OD a treatment is available [1]. The search of possible targets and strategies for OD therapy is very actively pursued by basic science. However, translating the resulting knowledge into new drugs is complicated by the high costs of pre-clinical and clinical research, in comparison with the small potential for revenue to pharmaceutical industry, because of the small market size [1]. Drug repositioning, i.e. finding new indications for approved drugs, represents to date one of the most cost- and time-effective strategies to identify a therapy for at least a subset of OD [2]. The rational basis for drug repositioning is given by the complex relationships between disease phenotypes, their underlying molecular mechanisms and the drugs that target them [3,4]. Indeed, it is increasingly recognized that the instances in which a clinical syndrome can be associated to a single molecular mechanism, which can be targeted by a very specific drug, is the exception rather than the rule in clinical practice. In particular, on one hand rare diseases and common disorders very often share some of their clinical features and underlying pathogenetic mechanisms [3,4]. On the other hand, the pathogenetic mechanisms of rare and common disorders often impinge on the function of entire molecular networks or functional modules, rather than on the function of single genes [3,4]. Finally, most of the chemical compounds approved for therapy are capable to bind and modify multiple molecular entities, producing a complex mixture of on-target and off-target effects [5]. On the basis of this complexity, it is not surprising that in many cases a pharmacological compound originally approved for a common disorder has turned out to be useful to treat phenotypically related, or even apparently unrelated rare diseases, and vice versa [2,6,7]. So far, these discoveries have mostly been achieved serendipitously through the clinical monitoring of drug effects. However, in recent years, the availability of huge information on the genetic basis of human disorders, on gene regulation, on protein structure and on drug-target interactions has generated unprecedented opportunities to pursue drug repositioning on a more rational ground [8]. Accordingly, several computational approaches have been developed to discover unrecognized or non-explicit connections between drugs, targets and diseases. Many of these strategies have used structural similarity between ligands and target proteins, together with literature mining and protein-protein interaction maps, to produce wide drug-target networks, allowing to infer new potential drug-target associations [8-10]. Genome wide association studies have been used with the same purpose [11]. Gene expression data represent a very rich alternative resource for inferring non-obvious relationships between drugs and drug target genes, which can potentially be used for drug repositioning [12-14]. This approach is based on the commonly accepted principle that genes implicated in the same functional modules tend to display very similar expression patterns under physiological conditions and after internal or external perturbations. The strongest initiative in this sense is the Connectivity Map (CMap), a collection of genome-wide transcriptional expression data from cultured human cells treated with bioactive small molecules that allows the discovery of functional connections between drugs, genes and diseases through the analysis of common gene-expression changes [15,16]. A network-based analysis of this resource has allowed to predict similarities in drug effect and mechanism of action, and to “reposition” a Rho-kinase inhibitor as an enhancer of cellular autophagy [13,14]. Another interesting example was the identification of ARA-C as a possible drug for Ewing sarcoma, based on the finding that the administration of this molecule to tumor cells negatively modulates a EWS/FLI oncoprotein-dependent gene signature [17]. Nevertheless, the potential of gene coexpression for proposing new associations between diseases, drug targets and drugs is still largely unexplored. The main limitation of the current approaches is that they mostly require gene expression profiles directly relevant to the condition under study, such as those obtained from patient cells and/or from suitable experimental models [13,16,17]. Unfortunately, these resources are not available in the majority of rare diseases. In this work we have developed a new computational strategy that may in part overcome this limitation. Our approach is based uniquely on the search for conserved anti-correlation between known drug targets and human disease genes, performed on public microarray databases. On this basis, we propose new potential candidate drug targets and drugs for rare human diseases for which no specific gene expression data are available.

## Results and discussion

### Generation and characterization of Conserved Anticorrelated Gene Clusters (CAGC)

**Figure 1:**
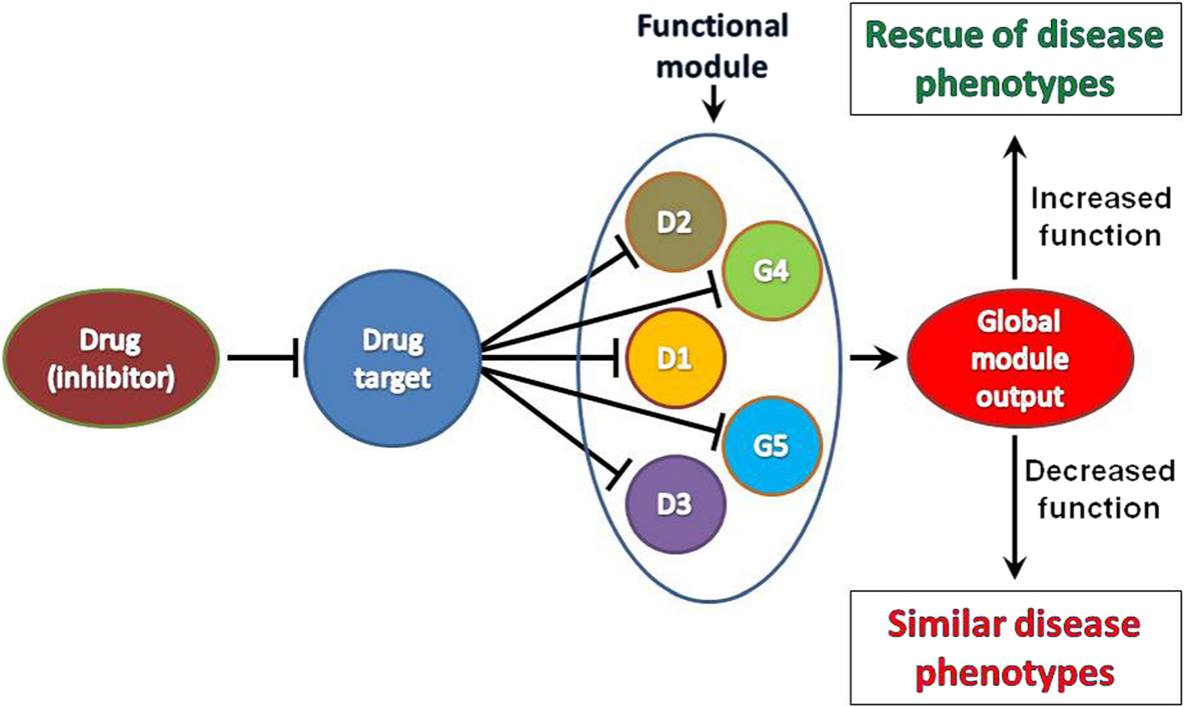
Schematic representation of how the antagonism between a drug target and a functional module containing functionally correlated genes, which may be implicated (D) or may be not implicated (G) in similar disease phenotypes, could be exploited to modify the outcome of genetic mutations.

**Figure 2:**
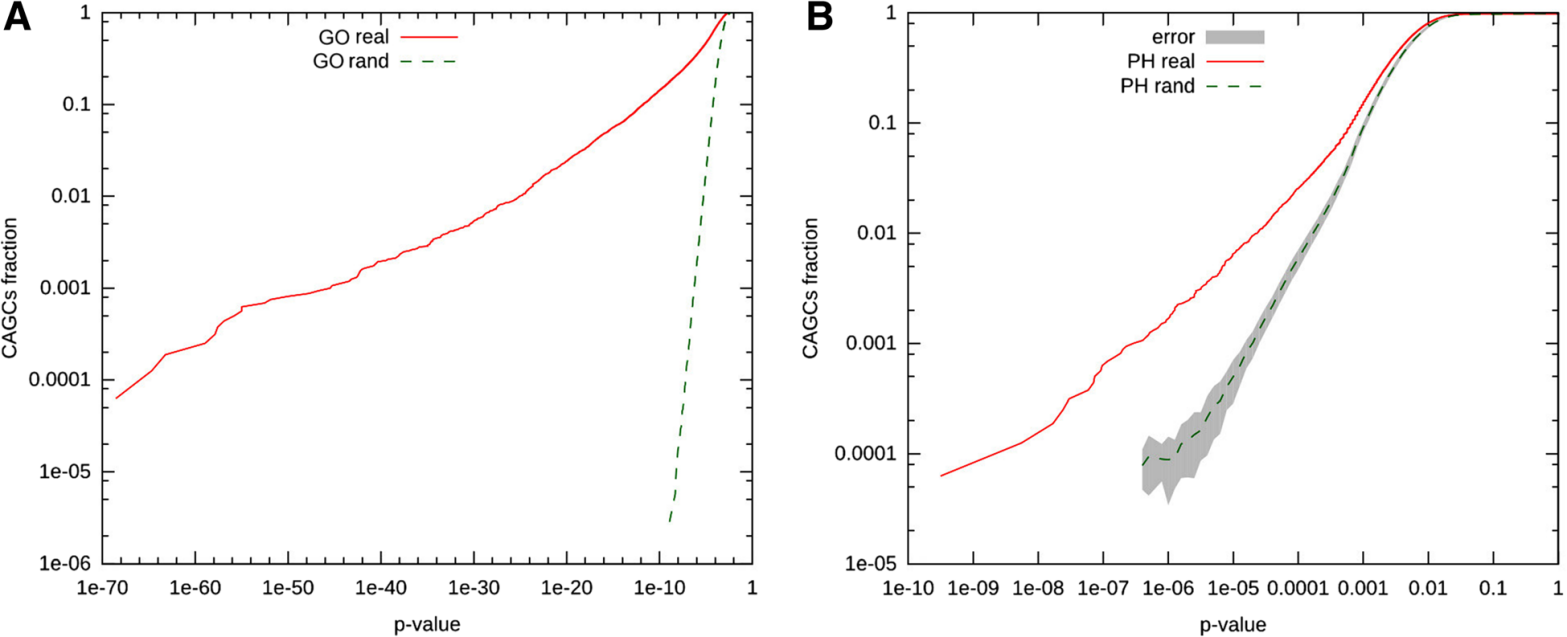
**Cumulative distribution of functional index for GO categories and OMIM phenotypes.** Cumulative log-log distribution of functional index (see methods) for **A)** GO categories, **B)** OMIM phenotypes (PH). Continuous curves represent the real data, while dashed curves result from 100 random permutation of real data (rand). Grey band in B corresponds to the standard deviation of the 100 randomly permuted datasets. The same band is not reported for panel A, since its thickness is comparable to the line thickness. In both cases the difference of real and permuted data is highly significant (P-value < 10e-256 Kolmogorov-Smirnov test).

Dissection of the molecular basis of many diseases has revealed that abnormal phenotypes are caused in most cases by the derangement of entire functional modules, due to single gene defects, to a combination of genetic abnormalities or to the interaction between gene variants and environmental factors [18,19]. The correct organization of these modules depends on a delicate equilibrium between positive and negative regulatory interactions. Positive functional interactions within a functional module are very often reflected by a highly similar expression profile (coexpression) of the genes that compose it. Indeed, it has been widely shown, through the systematic analysis of gene coexpression, that genes displaying very similar expression profiles tend to be functionally correlated [20] and that mutations affecting coexpressed genes tend to produce similar clinical syndromes [21-23]. Importantly, it has also been shown that these correlations are much stronger for genes that are consistently coexpressed across different species (conserved coexpression) [21,23-25]. Negative correlations have been much less investigated under this perspective. However, it has been suggested that if two genes display strongly anti-correlated expression profiles they may act in opposing functional modules or they may play opposing roles within the same functional module [26-28]. Potentially, this scenario is of great pharmacological relevance, because it would imply that the overall output of some disease-relevant functional modules could be positively or negatively modified by modulating the function of anti-correlated genes (Figure 1). Therefore, we decided to systematically analyze whether groups of mammalian genes displaying consistent anti-correlation with a specific gene are functionally characterized and are associated to similar disease phenotypes. To this aim, we studied a large, manually annotated microarray dataset downloaded from the Gene Expression Omnibus [29], covering many tissues, cell types and experimental conditions in both human (5188 experiments) and mouse (2310 experiments) [30]. We built generic and tissue-specific CAGC using a ranking-based procedure, very similar to the one that we previously used to identify conserved coexpression clusters ([21,30], see materials and methods for the details). In synthesis, the CACG for any particular gene of interest (*g*), referred to as the centre, is composed of the genes that fall in the bottom 1% correlation rank of *g* in both human and mouse (a cluster does not includes the centre itself). We then compared the Gene Ontology (GO) functional annotation [31] of the obtained clusters with the annotation of random clusters of same size. Genes composing CAGCs were strongly enriched for genes annotated to the same GO keywords (Figure 2A). This was an expected result [20,25,30], since genes composing a CAGC display strongly correlated expression profiles (data not shown). Interestingly, for clusters that showed at least one GO keyword enriched with a Bonferroni corrected p-value of 0.05 or less, 44% of the centre genes were annotated to at least one of the significant keywords. In addition, and most importantly, we found that the CAGCs are significantly enriched for genes associated to genetic diseases characterized by similar phenotypes (Figure 2B), as defined by MimMiner [32]. However, when in these cases the centre genes were also associated to mendelian disorders, we found that the MimMiner scores calculated between the disease associated to the centre gene and those associated to the other genes of CAGC never reached the 0.4 similarity threshold. Altogether, these results indicate that, although the genes that compose a CAGC and the centre of the same clusters frequently work in the same functional modules, the phenotypic consequences of a mutation of the centre gene are different from the consequences of a mutation of the genes composing the corresponding CAGC. This scenario is compatible with the idea that the genes of a CAGC and its centre gene may play functionally antagonistic roles.

### GAGCs centered on drug targets are significantly modulated by treatment with the relevant drug

**Figure 3:**
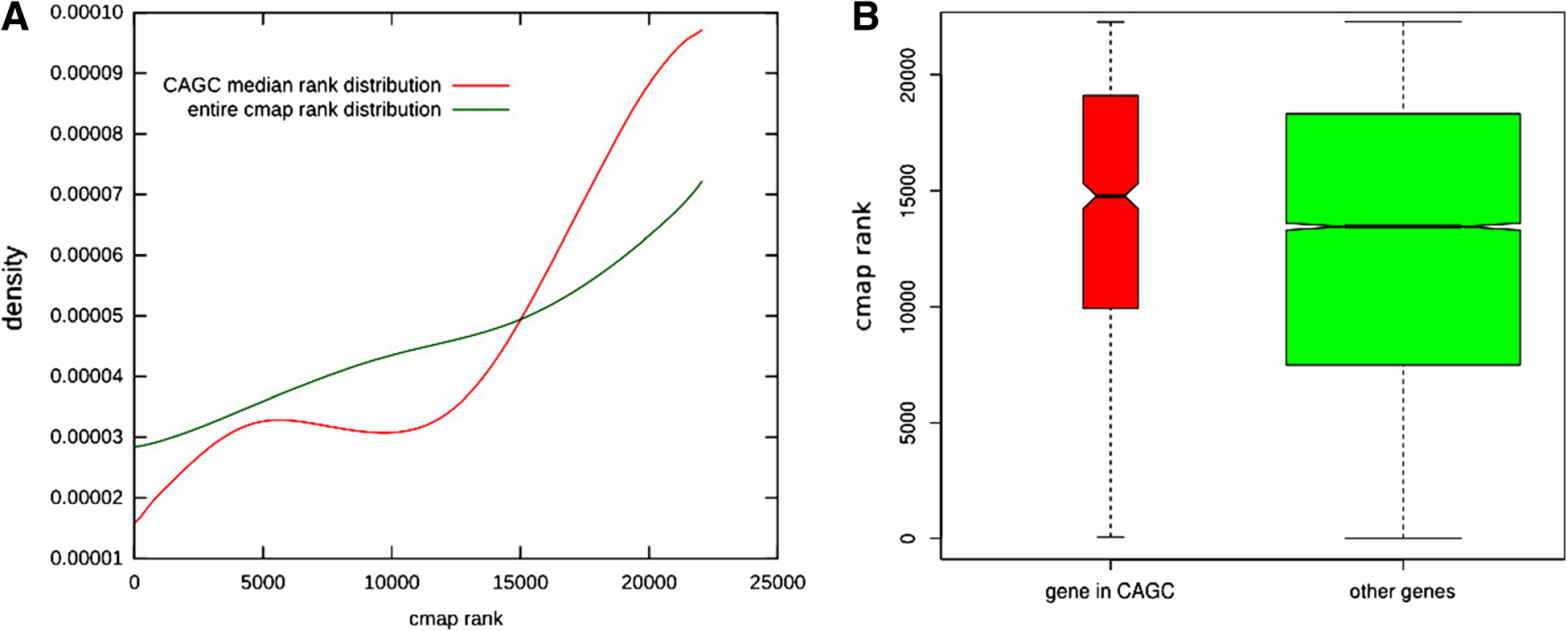
**Cmap rank in CAGC genes.** Panel **A** shows the gene rank distribution for all genes and all cmap instances (green curve) and the distribution of the median gene rank of each CAGC whose center is a known drug target (red curve). Note that the gene rank is defined as the highest rank among the probesets associated to the gene, which explains the non-uniform distribution of all genes. As an exemplar case, in panel **B** we show a box-plot comparing, for the cmap instance n. 436 (1e-05 M of Fasudil for 6 h in PC3 cells), the genes in the CAGC of ROCK1 (a known target of Fasudil) with all the other genes. The rank of the CAGC genes is significantly higher (Pvalue < 3.4e-05 Mann-Whitney U test), implying that the inhibition of the CAGC center by the drug leads to a general overexpression of the genes that compose its CAGC.

We then asked whether pharmacological modulation of CAGCs centres may actually lead to a modulation of the anticorrelated genes. To this aim, we analyzed the gene expression data contained in CMap, which were obtained from cultured human cells treated with bioactive small molecules [15,16]. Moreover, we obtained the definition of genes as targets of specific drugs from analysis of the DrugBank database [33]. For every CAGC centered on a drug target associated to a molecule used in CMap (682 associations), we tested whether the genes in the CAGC are significantly upregulated or downregulated upon drug treatment (Mann-Whitney U test). We performed 8435 tests, 3510 of which were significant with a False Discovery Rate (FDR) < 0.05. Importantly in 3428 cases the median ranks are consistent with an upregulation of the CAGC genes, while only in 82 cases it is consistent with a downregulation. The median rank distribution for all the CMap instances and the exemplar case of the genes composing the CAGC of ROCK1 (a target of Fasudil) in the CMap instance n. 436 (1e-05 M of Fasudil for 6 h in PC3 cells) are reported in Figure 3. Considering that the majority (76%) of the drugs used in CMap and associated to a cluster significantly up- or down-regulated are classified as inhibitors in DrugBank, these results strongly validate the general idea that the genes anticorrelated with a drug target are upregulated when the function of the target gene is inhibited.

### Association of available drug targets to orphan genetic disorders through CAGCs

**Figure 4:**
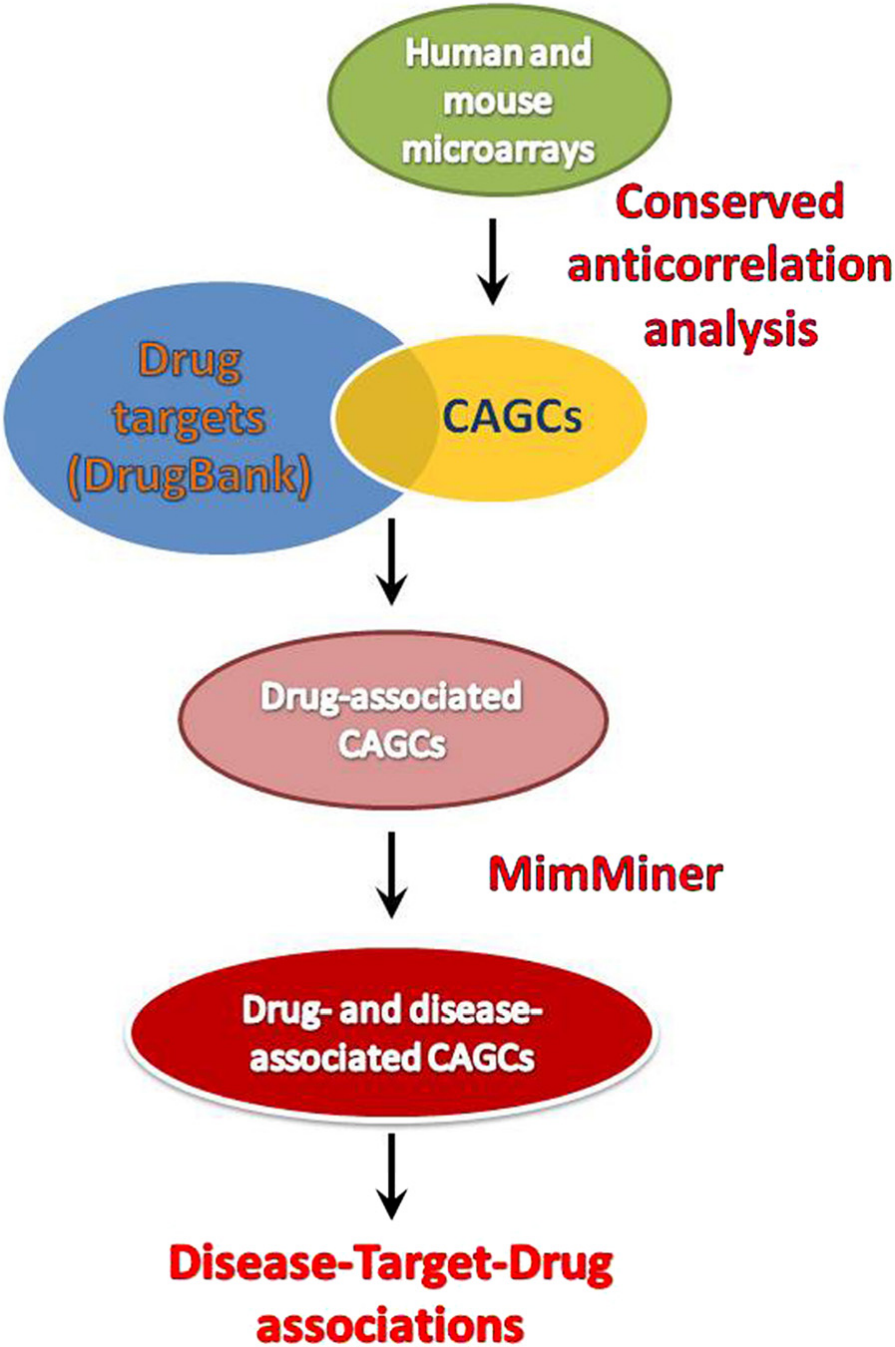
Schematic representation of the CAGC-based drug-repositioning pipeline.

On the basis of the above results, we thought to use CAGCs to associate know drug targets to mendelian genetic disorders. To this aim, we selected CAGCs characterized by two features: the centre of the CAGC is a drug target and the other genes that compose the cluster are significantly enriched for genes associated to related morbid map phenotypes [32] (Figure 4). Indeed, we would expect that the pharmacological down-modulation of some of these centres may compensate for the loss of at least some of the specific disease genes that are among them. Under this assumption, the drugs that are capable of inhibiting the CAGC centre would become potential candidates for all the related mendelian disorders associated to the cluster. In other words, we propose a drug as possible treatment for a genetic disease when the responsible gene (DG) belongs to an anticorrelation cluster that has the drug target as the centre and that is significantly enriched for other genes implicated in disorders similar to the disorder resulting from DG mutation. Using this strategy, we obtained the list of drug targets/disease associations reported in the Additional file 1. The list could contain both completely novel associations or associations which are already supported by previous evidence. Although we would expect most of the associations to be novel, finding a significant number of associations supported by literature could represent an important validation of our method. In consideration of the difficulty of automatically assessing this point, we decided to systematically inspect the list by expert analysis. The most prominent results are reported below.

– Calmodulins and malformative genetic syndromes

– Calmodulins 1 and 3 (CALM1 and CALM3) are, potentially, the most prominent targets underscored by our approach. Indeed, they are represented in 426 of the associations reported in the Additional file 1, on a total of 2973. Since the two genes are strongly coexpressed (data not shown), the corresponding CAGCs were largely overlapping. Interestingly, these CACGs were strongly enriched for genes implicated in very complex and heterogeneous genetic syndromes. However, although many of these disorders are characterized by abnormalities of skeletal development, considering the high number of involved genes, the crucial role of calcium/calmodulin in many different cellular processes and the high number of drugs associated in DrugBank to Calmodulins, we consider this as a likely non-specific result of our analysis, which underscores the potential pitfalls of our method.

– Drug targets for epileptic syndromes

– Epileptic seizures represent a frequent neurological condition, affecting approximately 3% of the population and represent one of the most common symptoms of genetic disorders affecting the Central Nervous System (CNS). Accordingly, many diseases represented in our list are characterized by epileptic seizures (Additional file 1). Interestingly, most of these disorders have been linked by our pipeline to at least two strongly validated drug targets: histone deacetylase 1 (HDAC1) and translocator protein (18kDa) (TSPO). HDAC1 is one of the targets of valproic acid, one of the most prominent anticonvulsant agents currently used in chronic epilepsy management [34]. TSPO, a conserved mitochondrial protein implicated in cholesterol transport and biosynthesis, is one of the targets of some benzodiazepines, which represent another prominent pharmacological class in the management of seizures [35,36]. In particular, although the most prominent targets of benzodiazepines are GABA receptors, it has been proposed that TSPO-binding compounds may contribute to anticonvulsant effects by regulating the synthesis of neurosteroids [35]. Another interesting gene highlighted by our analysis for the same syndromes was the prolyl-4-isomerase beta (P4HB). Indeed, although this case is by far less validated, if compared to the previous two proteins, P4HB has recently been proposed as a target for new antiepileptogenic drug design [37].

– Drug targets for autism spectrum disorders

– Besides to epileptic syndromes, HDAC1 was identified as possible target also for the autism-related disorder AUTS10 (MIM: 611016). We consider this result at least partially validated by the recent finding that HDAC1 inhibitors ameliorate social cognition and cell adhesion molecule plasticity deficits in a rodent model of autism spectrum disorder [38]. Similarly, we consider the association between prostaglandin-endoperoxide synthase 1 (PTGS1) and AUTSX2 partially validated by the finding that significantly elevated levels of prostaglandin E2 (PGE2) have been found in autistic patients [39].

– Drug targets for heart failure syndromes and for neuromuscular disorders

– The Ser/Thr kinase ROCK1, a downstream effector of the cytoskeletal modulator RhoA small GTPase [40], was associated by our pipeline to 57 different genetic disorders, characterized by a high prevalence of muscular, cardiac and neurodegenerative phenotypes (Table 1).

– Interestingly enough, specific inhibitors of this protein, such as the FDA approved Fasudil, have displayed beneficial effects in experimental animal models of some diseases included in the list. Indeed, ROCK1 has been shown to play an important role in the transition from cardiac hypertrophy to failure in mice [41] and the administration of ROCK1 inhibitors has repeatedly been associated to beneficial effects in non genetic models of heart failure [42-45]. Even more strikingly, it has been recently shown that Fasudil improves survival and promotes skeletal muscle development in a mouse genetic model of spinal muscular atrophy [46], one of the prominent diseases included in our list. Moreover, it is interesting to notice that ROCK1 expression has been correlated to disease progression in an animal model of ALS [47], although in these case a beneficial use of ROCK inhibitors has not been so far reported. A beneficial activity of ROCK inhibitors has also been documented in experimental models of myopathies. In particular, in muscle of dystrophin/utrophin double-knockout, which represent an intensely studied model of Duchenne muscular dystrophy, the RhoA/ROCK1 pathway is hyperactive, leading to a reduction of the myogenic potential of muscle stem cells [48]. Accordingly, treatment with a ROCK1 inhibitor improves the myogenic potential of stem cells and increases muscle regeneration in vivo [48]. A second interesting target for muscular disorders is given by the IKBKB (IKK-β) protein, a Ser/Thr kinase that phosphorylates and inactivates inhibitors of the NF-kB pathway [49] and which we found as potential target for four different myopathies. Indeed, not only it is well known that the NF-kB signalling is abnormally activated in myopathies such as Duchenne muscular dystrophy [50] and in limb-girdle muscular dystrophy [51], but it has been proved that NF-kB activation may functionally contribute to muscle degeneration [52]. Accordingly, it has been recently shown that NF-kB inhibition may improve muscle pathology and regeneration in the Mdx mouse model [53,54].

**Table 1:** List of the genetic disorders associated to the ROCK1 target

**OMIM ID**	**Disease name**	**P-value**
611126	Deficiency of a Acyl-coa dehydrogenase family, member 9.	1.5e-05
201450	Deficiency of acyl-coa dehydrogenase, medium-chain.	4.4e-05
201470	Deficiency of acyl-coa dehydrogenase, short-chain.	2.2e-05
201475	Deficiency of acyl-coa dehydrogenase, very long-chain.	4.2e-08
608810	Alpha-b crystallinopathy.	1.5e-05
205200	Amyotrophic lateral sclerosis, juvenile, with dementia.	5.1e-05
600996	Arrhythmogenic right ventricular dysplasia, familial, 2; arvd2.	7.9e-05
609160	Arrhythmogenic right ventricular dysplasia, familial, 7.	1.3e-05
604377	Aardioencephalomyopathy, fatal infantile, due to cytochrome c oxidase.	4.7e-09
602067	Cardiomyopathy, dilated, 1f; cmd1f.	5.9e-05
192600	Cardiomyopathy, familial hypertrophic 1; cmh1.	7.4e-05
115210	Cardiomyopathy, familial restrictive, 1; rcm1.	7.1e-05
212350	Cataract and cardiomyopathy.	4.6e-07
601253	Caveolin 3; cav3.	7.8e-05
609060	Combined oxidative phosphorylation deficiency 1; coxpd1.	6.3e-05
610505	Combined oxidative phosphorylation deficiency 3; coxpd3.	4.4e-08
611719	Combined oxidative phosphorylation deficiency 5; coxpd5.	9.9e-07
300257	Danon disease.	6.1e-06
602668	Dystrophia myotonica 2; dm2.	3.6e-05
158900	Facioscapulohumeral muscular dystrophy 1a; fshmd1a.	2.4e-05
229300	Friedreich ataxia 1; frda.	5.5e-05
253800	Fukuyama congenital muscular dystrophy; fcmd.	8.8e-05
232300	Glycogen storage disease ii.	4.9e-06
232400	Glycogen storage disease iii.	5.3e-06
261740	Glycogen storage disease of heart, lethal congenital.	5.7e-06
261670	Glycogen storage disease x; gsd10.	4.4e-06
600737	Inclusion body myopathy 2, autosomal recessive; ibm2.	8.8e-05
167320	Inclusion body myopathy with early-onset paget disease and frontotemporal.	5.3e-05
147421	Inclusion body miositis.	2.4e-05
606183	Laryngeal abductor paralysis with cerebellar ataxia and motor neuropathy.	1.9e-05
220111	Leigh syndrome, French-Canadian type; lsfc.	7.8e-05
300322	Lesch-nyhan syndrome; lns.	8.9e-05
109150	Machado-joseph disease; mjd.	8.1e-05
248800	Marinesco-sjogren syndrome; mss.	7.9e-05
252011	Mitochondrial complex ii deficiency.	6.7e-06
609560	Mitochondrial DNA depletion syndrome, myopathic form.	3.4e-08
600462	Mitochondrial myopathy and sideroblastic anemia; mlasa.	7.2e-05
500002	Mitochondrial myopathy with diabetes.	3.1e-06
251950	Mitochondrial myopathy with lactic acidosis.	2.5e-05
610773	Mitochondrial phosphate carrier deficiency.	5.5e-08
310200	Muscular dystrophy, duchenne type; dmd.	9.5e-05
605809	Myasthenia, familial infantile, 1.	2.3e-07
610542	Myasthenia, limb-girdle, with tubular aggregates.	4.9e-06
254210	Myasthenic syndrome, congenital, associated with episodic apnea.	5.2e-07
255125	Myopathy with lactic acidosis, hereditary; hml	3.9e-07
609500	Myopathy, autophagic vacuolar, infantile-onset.	3.6e-05
609200	Myotilinopathy.	2.6e-05
258450	Progressive external ophthalmoplegia with mitochondrial dna deletions.	1.2e-05
609286	Progressive external ophthalmoplegia with mitochondrial dna deletions.	2.1e-06
212138	Solute carrier family 25 (carnitine/acylcarnitine translocase), member.	4.3e-06
103220	Solute carrier family 25 (mitochondrial carrier, adenine nucleotide.	3.9e-06
604360	Spastic paraplegia 11, autosomal recessive; spg11.	9.4e-05
610250	Spastic paraplegia 31, autosomal dominant; spg31.	8.4e-05
183020	Spinal muscular atrophy, segmental.	7.7e-05
253300	Spinal muscular atrophy, type i; sma1.	6.2e-05
609015	Trifunctional protein deficiency.	2.9e-06
222300	Wolfram syndrome 1; wfs1.	7.5e-05

P-value refers to the enrichment for phenotypically correlated genes detected in the corresponding CAGC.

## Conclusions

We have provided proof of principle that the analysis of anti-correlated gene expression profiles could be systematically used as an innovative approach to explore the complex space of the interactions between genes, drugs and disease phenotypes. Moreover, we have implemented a predictive strategy capable to propose new associations of available drug targets and drugs to orphan genetic disorders. We have applied this approach to produce a list of such associations, which we provide as an open source of experimentally testable predictions, to be validated in cellular or animal disease models. On the basis of the validated or partially validated examples, it would seem to us reasonable to anticipate that, at least in some of the proposed cases, the new associations will turn out to be relevant not only to explore the possible use of the proposed drug targets in such disorders, but also to address new possible molecular mechanisms leading from gene inactivation to disease phenotypes. As a final remark, although we have only shown how this principle can be applied to the problem of drug repositioning, we can envisage that similar approaches could be used to help addressing other outstanding problems of drug development, such as predicting the possible side effects of inhibiting or stimulating new drug targets.

## Methods

### Definition and evaluation of CAGC

We studied a large, manually annotated, microarray dataset downloaded from the Gene Expression Omnibus (GEO), based on Affymetrix Plus 2 platform, covering many tissues, cell types and experimental conditions in both human (5188 experiments) and mouse (2310 experiments) [30]. Following procedures and considerations similar to those previously described in [21] we built tissue-specific conserved anti-coexpression networks. We first generated single species anti-coexpression gene networks (SAN) and then integrated them on the basis of human-mouse orthology. SANs were generated by first calculating the Pearson correlation coefficients of every microarray probeset with all the other probesets. A directed edge was established from probeset p1 to probeset p2 if p1 fell within the top 1% probesets in terms of anti-correlation with p2. These directed networks were then converted into undirected SANs by requiring a reciprocal <1% ranking. We mapped each probeset to corresponding Entrez Gene identifiers using the Affymetrix na26 annotation, then an undirected edge was established between two Entrez gene G1 and G2 if there was at least one edge from a probeset assigned to G1 to a probeset assigned to G2 and vice versa (reciprocal 1% ranking). We then constructed conserved anti-coexpression gene networks (CAGN) starting from SANs by mapping every Entrez Gene identifier to the corresponding Homologene cluster (build 63). Two human genes G1h and G2h were connected in the CAGN if 1) they were connected in the human SAN, 2) booth of them were associated one-to-one to mouse genes by Homologene and 3) the corresponding genes G1m and G2m were connected in the murine SAN.

We constructed 18 CAGNs: 15 are specific of a single tissue (Adipose tissue, Brain, Breast, Central Nervous System, Sensory ganglia, Gastrointestinal tract, Heart, Kidney, Liver, Lung, Lymphatic tissues, Ovary, Prostate, Skeletal muscle, Stratified Epithelium), meaning that both the human and the murine SAN contributing to the tissue specific CAGN are based on samples derived from the same tissue. The three remaining CAGNs are not tissue-specific, because one is based on all the samples contained in the database, the second is based on the samples deriving from all the normal human and mouse tissue while the third derives from tumor cells data. We have previously shown [30] that coexpression information deriving from various tissue-specific datasets and from heterogeneous non-tissue-specific datasets contains highly complementary information. We thus chose to merge all the 18 CAGNs into a single one that we used for subsequent analyses (the merged network contained 15954 genes). Finally, for each gene G present in the merged CAGN, we define a conserved anti-coexpression gene cluster (CAGC), composed by the nearest-neighbours G' of the gene G in the merged CAGN. The gene G, the centre of the cluster, is not considered as a member of the cluster. CAGC are available as Additional file 2 on the journal web site and at the URL http://www.cbu.mbcunito.it/ts-coexp.

#### Pheno-Clusters

Genetic disease phenotypes described in OMIM were correlated on the basis of MimMiner [32]. MimMiner assigns a similarity score to all pairs of OMIM phenotype records, based on the text mining analysis of their phenotype descriptions. The pheno-cluster of the phenotype P is the set of genes directly associated by OMIM to P or to a phenotype P' whose similarity with P is at least 0.4. The reason for choosing this cutoff is that biologically meaningful relationships were mostly detected in phenotype pairs with a similarity score equal to or greater than this value [32].

#### Drug-phenotype association

We statistically associated drug targets to phenotypes by evaluating the overlap between the CAGC and Pheno-Clusters, through a Fisher exact test. We considered as significant the overlaps with P value < 1e-4, corresponding to 2.5% Benjamini–Hochberg FDR.

We used as reference for known drugs and targets DrugBank version 3 [33]. If a drug targeted a CAGC centre and there was a statistically significant association between the CAGC and one or more phenotypes, we putatively inferred an effect of the drug for these phenotypes. The idea under this inference criterion is that, if a drug D inhibits its targets, D should increase the expression of the CAGC centered in G with an effect on the associated phenotypes. If this inference criterion is valid, then we can predict that a drug will increase the expression of CAGCs centered in its targets. To validate this observation we used gene-expression profiles derived from the treatment of cultured human cells with a large number of perturbagens produced by the CMap project (build 2) [16]. For the purpose of the current validation CMap can be viewed as a matrix having genes on the rows and experiments on columns. Each experiment consists of a perturbation of a cell-line with a given concentration of a drug.

The matrix values are properly rank-transformed ratios between normalized expression values of the gene in the treated cell-line and a control; the greater the value the greater the fold-change in expression of the gene in the case versus the control. CMap reports the data in terms of Affymetrix probesets, which we mapped to Entrez Gene using the standard Affymetrix annotation (version na32). Since many probesets can be associated to the same gene, in each CMap instance we define the gene rank as the rank associated to the probeset with the highest rank. This explains why the gene rank distribution tends to be growing (see Figure 3) while the distribution of probeset rank is uniform by construction. Moreover some ranks are lost since some probesets can't be associated to any gene. Since DrugBank and CMap use different drug identifiers, we were able to associate CMap data to only 682 DrugBank compound. We used Chembl (version 12) [55] as a third source of drug information to map drug identifiers. Given a CAGC associated to a drug D, we expect that its genes are over-expressed in cell lines treated with the drug targeting the CAGC centre. We tested this hypothesis independently for each CMap experiment that uses the D (there are possibly many experiment using the same drug with different concentration or different cell-lines) with a two sided Mann-Whitney U test. For each drug we performed N x M tests, where N is the number of CAGCs whose centre G is targeted by D, and M is the number of experiments in CMap that involve D.

#### Functional enrichment statistical validation

To verify that CAGC tend to contain genes sharing the same biological function we defined two functional indices, 1) for GO categories, 2) for pheno-clusters.

Given a CAGC we used the Fisher exact test to compute the p-value of the enrichment of genes in each GO category (pheno-cluster). The GO (pheno-cluster) functional index of a CAGC is then the p-value of the most significant GO category (pheno-clusters).

We computed the functional indices for each CAGC in the real network and in 100 random networks obtained by permuting node (gene) labels, and finally we compared the cumulative distribution of functional indices derived from real versus randomized networks using a Kolmogorov-Smirnov test (see Figure 2). GO data [31] were obtained from the NCBI Entrez Gene site. All the associations were considered, including those inferred from electronic annotation (IEA).

## Competing interests

The authors declare no competing interests.

## Authors’ contributions

IM participated in the design of the study, performed most the analysis and participated to manuscript writing. UA produced the anti-correlation networks. PP and FDC conceived, supervised the study, were responsible of the expert analysis of data reported in the Additional file 1 and wrote the manuscript. All authors read and approved the final manuscript.

## Supplementary Material

Additional file 1: Table S1 List the associations between available drug targets and orphan genetic disorders obtained through CAGCs. Click here for file

Additional file 2 Compressed zip version of tab-delimited text file, Full list of the Conserved Anti-coexpressed Gene Clusters. Click here for file
